# Are We Ready to Really Hear the Voices of Those Concerned? Lessons Learned from Listening to and Involving Children in Child and Family Psychology Research

**DOI:** 10.1007/s10567-023-00453-4

**Published:** 2023-09-12

**Authors:** Anna Sarkadi, Maria Thell, Karin Fängström, Anton Dahlberg, Anna Fäldt, Anna Pérez-Aronsson, Georgina Warner, Maria Eriksson

**Affiliations:** 1 Child Health and Parenting (CHAP), Department of Public Health and Caring Sciences, Uppsala University, Uppsala, Sweden; 2 Department of Social Sciences, Marie Cederschiöld University, Stockholm, Sweden

**Keywords:** Child rights, Research, Participation, Involvement, Vulnerability, Parenting, Co-creation

## Abstract

A changing view of children, accelerated by the Convention of the Rights of the Child (UN in Convention on the rights of the child, UN Doc. A/RES/44/25, 1989, http://www2.ohchr.org/english/law/pdf/crc.pdf) has shifted the landscape of child and family research over the last few decades. Once viewed with low credibility and operating outside the interpretive framework of adult researchers, the rights-bearing child is increasingly recognized not only as having the capacity but also the right to participate in research. More recently, this movement has transitioned from the direct engagement of children as research participants—now considered commonplace, although less so for those who are structurally vulnerable—to the involvement of children in research design, review, conduct, and dissemination. Yet, both practical and ethical challenges remain. While children have the right to participation, they also have the right to protection. In this commentary, we set out to: (i) lay forth epistemic, child rights, and child sociology arguments for doing research *about*, *with* and *by* children and youth; (ii) recount our own journey of including children and youth in research to demonstrate the unique knowledge and insights gained through these approaches; and (iii) offer lessons learned on how to engage children and youth in research, including the involvement of structurally vulnerable groups.

Traditionally, research concerning children within child and family studies has been *on* children, using clinical or experimental observations and/or parent report measures. Increasingly, research *about* children has evolved, where children are included as participants/informants in studies, although young children, children with disabilities and ‘structurally vulnerable’ children tend to be less represented in research. The latter refers to children who are disadvantaged due to social inequalities associated with age, class, disability, gender, ethnicity/race, sexuality, sexual identity, migration or other characteristic/identity. This could be due to both practical barriers and ethical arguments, where the need for protection is argued to outweigh the right to participation. Research *with* children, where children and youth are involved in the research process, is even less common, but an exciting path moving forward. Research *by* children is the least common approach, where researchers take a back-seat and supportive role while children and youth conduct data collection, analysis, and research communication.

In this paper, we set out to: (i) lay forth arguments for doing research *about*, *with* and *by* children and youth; (ii) recount our own journey of including children and youth in research and give examples to demonstrate the unique knowledge and insights gained through these approaches; and (iii) offer lessons learned on how to engage children and youth in research including the involvement of structurally vulnerable groups as co-creators of research, thus pushing the boundaries of current practices.

In outlining arguments in section “Arguments for Doing Research *About*, *With* and *By* Children and Youth” of the paper for research *about*, *with* and *by*, as opposed to *on*, children we will describe the changing views of childhood, the theory of epistemic injustice, and the concept of voice. This will be followed by models of participation and how vulnerability and agency can be understood regarding child and youth involvement in research. In section “Our Own Journey of Including Children and Youth in Research” of the paper, we will give an account of our own journey moving from research *on* children, to *about* and *with* children and youth, and our current attempts to conduct research *by* children and youth. In section “Lessons Learned on How to Engage Children and Youth in Research”, we offer lessons learned and practical guidance for other researchers who are looking to involve children and youth in their work.

This paper is a commentary; it is not a formal position paper or a systematic review. Neither is it a critical review of existing literature involving children in family psychology research. It is, however, a reflection of a journey of a research group that went from classic parenting research with parent-reported measures on child behavior problems, to children and youth being a natural part of the everyday work at our lab, involved in study design, data collection and research communication. Yet, challenges exist and we are constantly learning ourselves. We believe that by sharing our most salient arguments, experiences, and lessons learned, others can create their own version of a research lab where children’s contributions can be welcomed and accommodated.

## Arguments for Doing Research *About*, *With *and *By* Children and Youth

### A Changing View of Children Highlights Children as Competent Contributors

A changing view of children and childhood may partly explain the increasing involvement of children in research. During the twentieth century, childhood as a concept was raised and problematized in a number of academic fields as well as in policy and practice. Towards the end of the century, what has come to be known as the sociology of childhood, introducing a paradigm of childhood emerged, in which the child is no longer seen as a passive subject, but has agency and is thus active in the creation of their own social life (James et al., 1998; Prout & James, 2015). The contribution of childhood sociology challenged more traditional views of children and childhood, showing, for example, how children’s ability to understand the consequences of their actions—or of their passivity—can exceed expectations based on chronological age (Liabo et al., 2018). How researchers view children and childhood has a direct impact on how research is conducted, as can be seen in how the research field has changed focus in recent decades (Kellett, 2010; Kellett et al., 2004; Larsson et al., 2018).

The Convention on the Rights of the Child (CRC) (UN, 1989), which distinguishes the child as a subject with rights of its own, has contributed significantly to the changed view of children. According to the CRC (UN, 1989), every child has the right to: express their views and have them taken into account in all matters affecting them; adequate information to enable them to express their views; and to be informed by adults of their rights so that they can exercise them. The rights-bearing child not only has the capacity, but also the right to participate in research studies, which in turn imposes obligations on adult researchers. A rights-based approach to participatory research with children implies that children have the right not only to be supported in expressing their views, but also in forming them (Lundy & McEvoy, 2017).

### Addressing Epistemic Injustice is a Moral Obligation

The passive research experience of children can be understood through the theory of ‘epistemic injustice’ (Fricker, 2007). The concept refers to a wrong done to someone in their capacity as ‘knowers’. It includes two forms of injustice: *testimonial injustice* and *hermeneutical injustice*. *Testimonial injustice* is when someone is perceived as less credible because of their social identity, causing them to be ignored, silenced or disbelieved. *Hermeneutical injustice* occurs when someone lacks words for their own experiences, or their experiences are not well understood by themselves or others, because of the systematic exclusion of some groups from activities such as scholarship. Fricker (2007) gives examples of epistemic injustice related to identities such as gender, race and class, but scholars have later argued that children as a group experience epistemic injustice with severe consequences for them (Baumtrog & Peach, 2019).

Carel and Györffy (2014) argue that children are hermeneutically disadvantaged within the healthcare systems since these are adult-governed and thus foreign to children’s interpretative frameworks. The authors call for health professionals and other adults seeking to understand children to attempt to enter their interpretative frameworks, and strive to understand their testimonies from within their own worlds**.** Using examples from forensic contexts alongside research on children’s competence and reliability as testifiers, Burroughs and Tollefsen (2016) argue that children are subject to testimonial injustice and urge for adults to mitigate this testimonial injustice by *actively listening to children*. Baumtrog and Peach (2019) examine three cases when children in contact with child protective services and healthcare died, and argue that this can—at least partly—be related to a lack of recognition of the children’s testimonies. They discuss how the children in these cases might have been rendered particularly invisible due to intersecting identities such as young age, ethnicity and sexuality. We argue that to reduce the harm experienced by children and youth in their capacity as knowers (testimonial injustice) and reduce the lack of knowledge and understanding of their realities (hermeneutical injustice), research should not be conducted *on*, but *about*, *with* and *by* children, irrespective of social identities.

### Models of Child Participation Exist to Help in Practicing Involvement

**Fig. 1:**
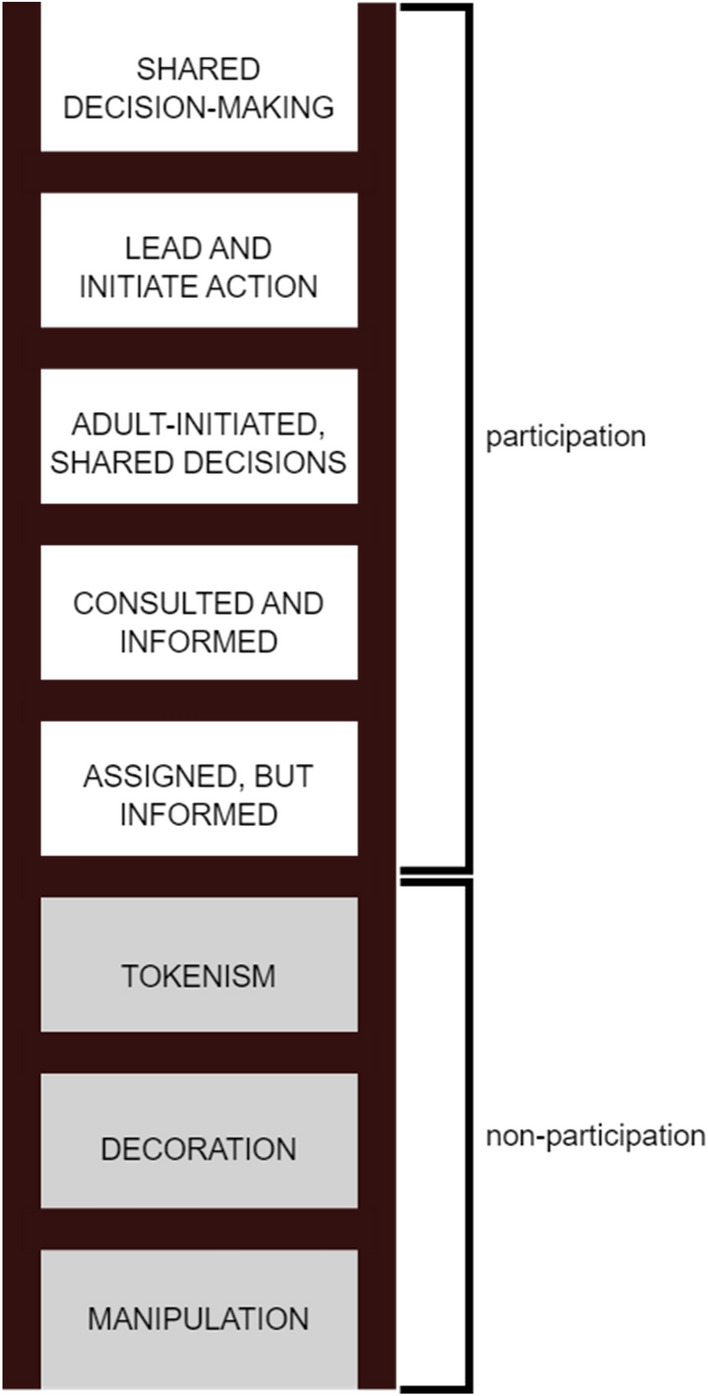
The ladder of participation (Hart, 1992)

In the wake of shifting views on children within both policy and research over the last decades there has been an ever-growing upsurge of publications discussing children and participation within research and beyond. Summarizing these debates, McMellon and Tisdall (2020) point to Hart’s (1992) “ladder of children’s participation” as an important point of departure in the research field (Fig. 1). The Hart model provided an early typology defining what constitutes participation in the CRC sense and what does not (e.g., symbolic or tokenistic ways of involving children). Furthermore, the model outlines different aspects of participation (i.e., information, consultation, decision-making, initiative) and levels of participation. Hart’s participation ladder has been frequently cited in the literature as well as used in practice to consider how children and young people can be involved in decision-making. Possibly because of this extensive use, it has been subject to criticisms (McMellon and Tisdall 2020). While Hart (2008) has clarified the ladder is not intended as a model by which stepwise progression toward the top is expected, it has been questioned for its linear nature and hierarchal positioning of participation approaches. A number of alternatives and complements to Hart’s ladder have, therefore, been designed over the years.

One such complement, specifically designed for child participation in research, is a matrix tool that positions dimensions of child control against the various phases of a research project (Shier, 2019). The matrix design allows for variable levels of participation throughout the process. The tool can be used prospectively to plan a project, or retrospectively to reflect on child participation throughout a completed project. It is intended to support researchers in questioning at what stage of the research process children should get involved, how children could be involved, and which children could be involved. Shier (2019) highlights that it would be a mistake to view the matrix content as a set of norms or targets to strive for. Children’s involvement may, for several reasons, shift during the course of a research project and a higher level of involvement is not necessarily the best at all stages. However, asking questions about *how*, *when* and *who* throughout the process means that decisions on these issues will be consciously made.

Some of the early models of children’s participation have been criticized for presuming that participation is inherently good and for not taking into account issues of power or the potential that there may be unintended negative consequences within participatory projects. Efforts to overcome such shortcomings include, for example, the P7 framework outlined by Cahill and Dadvand (2018) as a “thinking tool” intended to aid the unpacking and addressing complexities associated with children’s participation, including within research. The P7 model directs the attention to (1) purpose, (2) positioning, (3) perspective, (4) power relations, (5) protection, (6) place, and (7) process of involving children. As is clear from the literature, efforts to provide tools to both practitioners and researchers tend to result in models or frameworks that are rather complex, and thus somewhat difficult to put into (research) practice. For example, Shier’s original model (2001) of pathways to child participation includes 15 different points to consider, at five levels of the pathway. However, there are also models that might be easier to put into practice (Kennan et al., 2019), such as the Lundy model (2007) that has four components: (1) space, i.e., children must be given safe, inclusive opportunities to form and express their view; (2) voice, i.e., children must be facilitated to express their view; (3) audience, i.e., children’s view must be listened to; and (4) influence, i.e., children’s view must be acted upon, as appropriate.

### The Concept of Voice—We Should Aim to *Listen *to, Not Just *Hear* Children’s Voices

Models such as the Lundy one brings another contested concept within the field of children’s participation to the fore—the concept of voice. Especially commentators drawing on post-structural perspectives have pointed out the need to theorize voice in research with children (see Facca et al., 2020 for an overview). Within this strand of thought, notions of an authentic voice residing within the child has been questioned, and commentators have argued that voice should be treated and accounted for as a complex, relational, construction where meanings are always situated and open to multiple interpretations. Furthermore, they argued that children’s voice is almost always produced through intergenerational dialogue that includes members of other age categories, including adults (Facca et al., 2020). These dialogues will also be shaped by the institutional context in which they take place (Iversen, 2012, 2019). Thus, factors that mediate child–adult interactions and relations such as context and power need to be considered, and “researchers should explicitly and reflexively attend to the methodological implications of their approach to voice, and its influence on how data are generated and analyzed and children’s contributions to the research are re/presented” (Facca et al., 2020, p. 9).

Without dismissing debates such as the one outlined above, instead bearing it in mind, we would still argue that there is a need for knowledge development through research placing children’s voices centrally. Drawing on another epistemological and methodological approach than a post-structural one, it could be argued that it is possible for adults and researchers to move beyond ‘hearing’ children, i.e., engaging in dialogue shaped by adult agendas, to ‘listening’ to the life world of children (Gräfe & Englander, 2022). As indicated by Heimer et al. (2018), when children are not given opportunity to influence the framing of what “the problem” is (or their need, we may add), the design of interventions tends to be poorly matched to the actual problems (or needs). A recent review of literature on children’s participation in developing interventions in health and well-being indicated that work remains in enabling children and young people to influence the development of interventions (Larsson et al., 2018). We also note that the level of participation varied from children and young people taking part just as active informants, through stages of greater participation both in quantitative and qualitative terms, to children and young people becoming an active agent involved as a collaborator where the research process was shaped by views of a higher level of mutuality. However, the latter was the least common approach found (Larsson et al., 2018). In addition, we know that this is the case especially when it comes to children considered to be ‘vulnerable’ (see e.g., Cody, 2017; Kay & Tisdall, 2012; Sandland, 2017). Thus, there is yet work to be done in becoming way better listeners to children’s voices.

### Do Not Let Vulnerability Get into the Way for Agency

The CRC (UN, 1989) is based on a caring perspective and, while the child has the right to participation, they also have the right to protection and guidance from parents and other adults around the child. The caring perspective has historically tended to take precedence, not least in research and particularly in relation to children who can be deemed ‘vulnerable’. This ‘vulnerability’ has then been used as an argument to exclude groups of children and young people from participating in research. Recent research from the biomedical field has challenged this by demonstrating that involving structurally vulnerable children and families in research through *partnerships* can in fact be protective (Sammons et al., 2016). So, rather than protecting children and young people *from* research, there are ways of protecting them *through* research.

Participatory research practices involving children engage with children’s vulnerability in multiple ways. Debates on this strand of research has to a large extent concerned children’s vulnerability due to the age order (Eriksson & Näsman, 2012). The notion of vulnerability in itself has been debated, as it has, on the one hand, been argued that it is a universal and inevitable condition of being human because we are embodied (Tisdall & Kay, 2017). On the other hand, the notion of vulnerability has also been discussed as paternalistic and oppressive, that it can be a very controlling and stigmatizing label rather than an emancipatory one. One problem, it is argued, is that it is the vulnerable position of children that tends to be focused upon, rather than power, and too little attention is given to the contextual and structural causes and too much attention to individuals who are vulnerable and/or dependent (Tisdall & Kay, 2017).

Some of the debates regarding the notion of vulnerability, for example when it comes to child welfare, are rather abstract and concern theorization of state-citizen relationships. Our take on issues of vulnerability and agency is another one, as we are more pragmatically interested in research involving children. If children participating in research experience a difficult life situation, in addition to the position of being a child, vulnerabilities have to be tackled. Drawing on previous debates and empirical research, we argue that *there is no necessary contradiction between recognizing these vulnerabilities and participatory research involving children*. Instead, the principle of care (for the vulnerable, dependent child) and the principle of participation (for the competent child) can be combined in a dual approach to children participating in research (Eriksson & Näsman, 2008, 2012). Using empirical studies on children exposed to intimate partner violence as a case in point, the research body developed over the last decades demonstrates the agency, competence and capacity of many vulnerable children. It shows how children are not ‘passive’ victims of situations with violence. Instead, they try to make sense of these experiences, and they attempt to intervene and manage both situations and relationships (Eriksson et al., 2022). Furthermore, these studies show that as there are limits to adult knowledge, involving children in research is necessary to gain insights into, for example, children’s emotions and under which conditions they feel safe and protected (regardless of whether adults think that they are safe and protected). In addition, children’s participation—in research as well as everyday life—may create opportunities for validating difficult life experiences, and thereby support children’s recovery. Thus, both the category of ‘vulnerable child’ and the notion of participation should be unpicked and the discussion needs to be more refined regarding how children—and in particular vulnerable children—can be included in research. Here our approach is in line with other models of how to enable participation for children in vulnerable life situations (e.g., Skyrme & Woods, 2018). For example, drawing on Mullender et al. (2002), Houghton (2015) argues that research with children in vulnerable life situations needs to consider both three Cs and Ds (consent, confidentiality, child protection: danger, distress, disclosure) of the Mullender et al. approach, and three Es focusing on children’s power and impact: enjoyment, empowerment and emancipation (see also Houghton, 2018).

In which ways the principles of care and participation can be balanced and combined in encounters with different groups of children (e.g., in terms of age or life situation), in different kinds of research, and in different steps of the research process will be discussed further in the section drawing on our own recent studies below.

## Our Own Journey of Including Children and Youth in Research

### Research on Children

By research *on* children, we mean a design where children are the focus of, but are not directly included as informants in research. We started our own research on parents and children using both survey (Salari et al., 2014; Wells et al., 2016) and interview methods (Rahmqvist et al., 2013). We got involved in parenting intervention research (Sampaio et al., 2015) and still have some ongoing work about implementing parenting programs (Dahlberg et al., 2022). While parents are the intervention target in such programs, the program logic is of course centered on child outcomes. We grew increasingly more interested in accessing children’s own views, especially preschool-aged children’s, who were the target of our parenting interventions. Yet, we had trouble knowing how to conduct such research.

## Research About Children

By research *about* children, we mean a design where children are directly included as participants or informants. It was at a parenting research conference that we were introduced to the computer-assisted interview for young children, In My Shoes. It is fair to say that it became a game-changer for our research group. In My Shoes is an interactive method, where the child and interviewer use the software together in a triadic interview. While not carrying out the actual interview, the computer program provides a visual aid, as well as structure, a shared external focus and tools for expressing emotional and relational experiences. A suit of studies followed to establish the feasibility and validity of the tool (Bokström et al., 2016; Fängström et al., 2016). Apart from now having a tool to access children’s voices we had gained a sense of the richness and crucial importance of children’s own experiences. When, for example, the new national guidelines for vision testing at the universal child health services were created, it was one of our In My Shoes studies, conducted on the 4-year universal child health visit, that provided important information about some children’s clear discomfort with occlusion bandages (Fängström et al., 2017).

Examples of research about children in the parenting field followed, not just using In My Shoes, but other data collection methods as well. We took the opportunity of a local science fair to explore children’s views of what constitutes good parenting. We asked children 4–14 years of age (*N* = 280) a single open-ended question: “What is a good parent?”. The most common characteristics of a ‘good parent’ were being *kind* (42%), *caring* (19%), and *fun*. Children also expressed clear ideas about the value of *limit setting* (10%), often acknowledging the need for *both* being caring and setting limits. A number of children explicitly said a good parent should *not hit or hurt* a child (3%) or should *not be angry or shout* (4%). The majority of the codes identified in this analysis related to a warm and caring parent–child relationship, well in accordance with the general emphasis of parenting programs on spending ample quality time with the child to build the relationship. The fact that assertive discipline, along with quality time seems to be the optimal combination in effective parenting programs (Kaminski et al., 2008) seemed to resonate well with children.

One year later at the same science fair, we elicited children’s views on “sharenting”, i.e., when parents share pictures or information about their children via social media. Children 4–17 years old (*N* = 68) answered an online anonymous picture-supported questionnaire about different forms of sharenting (Sarkadi et al., 2020a). They were most negative towards parents posting pictures of or writing things about their children on social media, but also towards taking a photo without permission. Children and youth wanted parents to ask them before taking pictures or sharing images of them and to listen to their answers. By asking children directly about this parenting practice, their frustrations have been voiced and can inform parenting guidance on the exposure of children via social media.

Our pursuit of the children’s perspective on parenting has continued. Most recently, we have explored a central social learning theory in parenting, namely the coercive cycle, from children’s perspective. Using the In My Shoes interview methods, children aged 3–5 years (*N* = 21) were asked about their experiences related to emotions and relationships in the home. The children were able to share rich accounts of how they and their parents became caught in cycles of coercion, with vivid descriptions of escalations and negative affect in both themselves and their parents. In some cases, parental hostility and violence were disclosed. There was also a general lack of descriptions of quality time between children and their parents. Even the youngest children were able to reproduce such sequences of events, both their own and their parents’ behaviors, in a clear manner (Dahlberg et al., in press).

Beyond intervention research, we believe children’s voices have a place in research about broader societal issues. Once again using the science fair setting, we engaged children on the topic of forced migration. While the development of immigration policy is an adult-governed arena, there is a growing body of research demonstrating that peers play a significant role in the promotion of refugee children’s well-being. It is therefore important to engage children, as important social actors, with the topic of forced migration and elicit their views on the matter. We did this through a film-based activity. A short clay animation film conveyed a typical experience of a refugee child to provide contextual knowledge, then children aged 5–14 years (*N* = 51) completed an anonymous, open-ended questionnaire about support needs (Sarkadi et al., 2022). The qualitative design was selected to give the children space to provide detail about their reasoning in their own words and to enable the nuanced perspectives of children across various ages to be captured. The responses formed four categories: ‘Practical support’, ‘Emotional support’, Social inclusion’, and ‘Policies’. Examples of responses were “Have a friend who understands” (Emotional support); “See and treat them like an ‘ordinary’ child… give them the same possibilities as for the other children” (Social inclusion); and “A little part of the tax could go to them” (Policies). The findings demonstrate that school-aged children can be engaged with the topic of forced migration, and have relevant ideas about the support needs of refugee peers.

When the pandemic hit, our first thought within the research group was how to capture children’s experiences of the unprecedented societal event. We launched a national anonymous web survey asking young people, aged 4–18 years (*N* = 1047), about their pandemic experience, including whether they were experiencing any worrisome thoughts (Sarkadi et al., 2021). Worry was common, but how the children expressed their worries varied by age with younger children utilizing story-like narratives and older children giving more complex responses. The nature of their worrisome thoughts also differed, with adolescents expressing greater concern regarding the future and wider society. In addition to asking children to express their pandemic experiences with words, we adopted an arts-based research method by analyzing their drawings. The analysis of drawings by 4–6-year-old children (*N* = 91) revealed their need to grasp the ‘invisible enemy’, the profound effects of the pandemic on their lives in Sweden, despite comparatively mild restrictions, and their surprisingly high level of health literacy regarding COVID-19 (Sarkadi et al., 2023).

We recognized that children living with disabilities were an especially vulnerable group during the pandemic, having both higher medical risks and at risk of social isolation. So, we conducted a qualitative study specifically targeting this group. Six children, 5–12 years of age living with a severe disability, were interviewed about their pandemic experiences (Fäldt et al., 2022). They reported feeling lonely and bored, missing their grandparents and spare time activities. Several of the children lacked a spoken language, so we used augmentative and alternative communication during the interviews. Many parents were surprised how well aware their children were of the pandemic and had not heard about their worries before the interviews.

### Research with Children and Youth

By research *with* children and youth we mean a design where they are involved in the research process, in collaboration with academic researchers. This can comprise involvement in any or all phases of the research, based on what is possible and desired by researchers and children, as described by Shier (2019). In another pandemic-related project, we worked with young people to explore how COVID-19 was being discussed on social media by people their age. We recognized that, while we had the necessary ethical clearance to access adolescents’ social media postings, young people were better suited to the task. More familiar with the social media landscape of adolescents, they knew where to look for the information and could filter it though their own pandemic experiences and thoughts. So, we engaged 23 youth, aged 13–19 years, in the data collection phase where they independently collected material from their social media related to COVID-19 and describe their reasons for the selection in field notes. Content analysis was used to explore the motives annotated in the field notes for choice of social media material. We believe their perspectives captured the essence of the social media data in a way we, as adult researchers, would not have been able to. The research highlighted not only that social media platforms were important tools in reaching out to the adolescent community during a societal crisis, but that youth should be involved in tailoring such crisis information and related decrees for their target group. It also seemed that for youth the possibility to discuss this type of information and be able to express and receive social support were just as important, which further emphasizes why they should be involved in co-producing such outlets (Lygnegård et al., 2023).

Again, during the pandemic period, we turned to youth for support with an issue we were facing in an interventional study. We were in the midst of evaluating a group-based trauma support program for refugee youth (Sarkadi et al., 2020b; Warner et al., 2020). While societal restrictions were relatively scarce in Sweden, secondary schools—our implementation arena—moved to distance learning via online platforms. If we were to follow suit and move our intervention online we wanted to do it right and recognized the need for a youth perspective on the matter. We formed a team consisting of three young people, 17–19 years old, with personal experience of trauma and forced migration, one intervention group leader, and two researchers, who worked together in a series of participatory workshops (Pérez-Aronsson et al., 2022). The young people were highly involved in planning the research process and in generating recommendations and resources for delivering the intervention online. A panel of parents and professionals reviewed the online delivery recommendations that the team created and, although they largely agreed, there were points of disagreement. For example, the youth placed high value on social aspects and flexibility, while the professionals emphasized safety aspects and intervention consistency. This illustrates the importance of allowing all relevant voices to be heard. Two of the youth continued to be involved after the adaptation process. They contributed to research dissemination by co-authoring the scientific paper and participating as panelists at a research conference. Drawing on their experiences of involvement in the research process, they also took the initiative to draft a set of recommendations for other researchers who want to involve youth in research (manuscript). Their long-term commitment and engagement show that youth can and want to be involved in research, and that they have important contributions to make. During the project, the youth identified the potential for a trauma app based on the intervention. This gave rise to another collaborative project in which we have been working with a group of seven young people, 14–19 years old, with personal experience of trauma to co-design an app. The young people have been highly active throughout the development process and made substantial contributions to the mechanics, dynamics and esthetics of the app.

### Research by Children and Youth

By research *by* children and youth we mean a design where they take responsibility for parts of or the whole research process. When research involves human subjects, specific ethical legislation and guidelines apply for which youth cannot be responsible. However, researchers can provide a context where the legal and security conditions are provided to enable youth to lead on parts of a project, with researchers taking a back-seat position from which they support and consult the youth. An example from our work is the current phase of the trauma app project, where the young people are responsible for data collection. They are conducting usability testing of the app with a non-clinical population after having received training from us; thus, data collection is being done *by* these youth. Subsequently, the data analysis and writing will be done in collaboration between the young people and academic researchers, i.e., *with* the youth. The process has been designed to allow the young people to be co-authors on the planned publication. The project has demonstrated that young people whom may be considered ‘vulnerable’ due to their lived experience of trauma are willing and amply capable to contribute to the design and research process.

## Lessons Learned on How to Engage Children and Youth in Research

A key message from debates on children’s participation in research, is the necessity of being reflexive, on how we as adult researchers engage with children and create preconditions for their opportunities of having a voice. Below we outline some approaches we have used to try to enhance children’s participation. We have highlighted the most important lessons learned in Table 1.

**Table 1:** Key lessons learnt in involving children and young people in research

Step in involving children in research	Things to consider
Recruitment	Carefully consider language and arena, piggyback local events or other outreach activities, use social media
Engagement	How can research activities be fun and challenging yet respectful of children’s time, schedules and other commitments? Are there opportunities for empowerment?
Consent	Information needs to be accessible, comprehensible and not overwhelming. Situational assent always applies. Parental consent regulations vary with country
Safeguards	Prepare for unexpected disclosures, distress or need of help. Have adequate safety protocols and routines for e.g., contact with child protection or youth mental health services
	Enable discussion of lived experiences without the need to disclose personal stories. Be prepared to involve child protection services
Participation in interviews	Age adequate structure to interviews. For young children, encouraging to talk only about things that happened, making sure there is a ‘stop’ sign for discontinuation, and that it is ok to say ‘I don’t know’ are important rules to share and practice. Using pictures or pictorial support and conducting interviews side-by-side can assist young children’s narratives
Participation in co-produced research activities	Co-create your working methods, pay a lot of attention to practical details, such as communication channels, time and place for meetings. Be VERY flexible and don’t judge when life gets in the way for young people. Invest time and effort in relationship building and train young people in their new role
Contribution	Children’s participation must be acknowledged, and their contribution recognized, through reimbursement or other means, including authorship when adequate

### Recruitment

It is important to give attention to *how children are recruited* to research studies. Child, parent, family and neighborhood characteristics have all been posited as factors affecting children’s involvement in research (Robinson et al., 2016). In research conducted with children and young people, challenges regarding gender representation are also apparent (Åkerström, 2014). While some factors may be difficult for researchers to influence, others can be actively considered when recruiting. For instance, enabling participation in various languages—as we did for our national web survey during the pandemic—can support the inclusion of children from linguistic minorities who are reportedly less involved in research (Robinson et al., 2016).

The recruitment *arena* is also key. Families with lower socioeconomic status (SES) tend to be less involved in research; lack of time and fewer resources to enable participation have been cited as causes (Robinson et al., 2016). By facilitating participation opportunities whereby parents do not need to commit time or resources to the process, children from lower SES families have a greater chance to take part. Our studies hosted at the children’s science festival serve as a good example. The science festival is well-attended by local schools; thus, participation is facilitated for many children in the area. Anonymous momentary data collection design, while it limits the ability to describe the sample in detail, enables children to choose for themselves whether they would like to take part and no additional time nor resources are required.

To recruit the team for the app project, we used a youth organization that works with a relevant target group for the project. However, not all young people are involved in organizations, for many different reasons. Therefore, we also contacted a large youth center in an area considered ‘vulnerable’ by the Swedish Police Authority, which, in addition to posting the recruitment material on its physical notice board, also posted it on its digital channels—reaching hundreds of young people. Most of the young people in the team found out about the project through the youth center and the process helped us reach a wider representation in terms of age, gender and other background factors.

### Engagement

Ethical aspects of research can be more pronounced with children and thus require greater awareness. It is for this reason that dedicated guidance has emerged, e.g., Ethics Research With Children (ERIC, 2016). Of course, we must minimize harm—but we must also consider how to provide benefit. When planning our research activities, we should consider how to make them enjoyable and how to provide learning and empowerment opportunities for the children involved. Even very vulnerable groups of children can have a profoundly empowering experience from participating in research, if adequately engaged and listened to (Liabo et al., 2018). Yet, we must remember that participating in research is fundamentally altruistic; it is an imposition, regardless of potential benefits.

### Consent

A fundamental ethical principle that deserves careful consideration is informed consent. We must strike a balance between conveying enough detail about the research to make a meaningful decision, while ensuring the information is accessible, comprehensible and not overwhelming. Situational assent always applies, especially with younger children. It is important to establish ‘stop signs’ and offer brakes, snack or multiple occasions for participation if fatigue gets in the way of participation. Parental consent regulations vary with country, in Sweden youth 15 and above can consent to research on their own behalf without parental consent.

Our group is currently evaluating how children and adolescents, between ages 3 and 17 years, experience and understand research information and consent forms. The first part of the study consists of usability testing of research information for child and youth participants that the Swedish Ethics Review Board has previously approved, specifically looking at the mandatory information parts on handling sensitive personal information and how participants perceive what exactly they consent/provide assent to. In the second part of the study we will work with children and adolescents to create research information specifically adapted to their needs and preferences regarding the above sections of the research information and consent forms. Finally, we will advocate for this co-produced information to make it to the Ethics Review Board’s recommended formulations for consent letters in studies involving children.

### Safeguards

Distress can emerge in research participants of any age, but there are further considerations when children are involved. For example, they may not have shared their experiences before, know where to get support, or understand the impact of sharing their memories or emotions. It is important for us as researchers to anticipate topics that might cause distress, whether it is possible to avoid distress, and to plan our research methods carefully to be able to monitor and respond to signs of discomfort.

Being prepared for having to contact child protection services is another safety issue. We always inform parents that we have an obligation to report to child protection services should we gain information that causes worry about the child’s safety and/or wellbeing. In fact, in the study on the coercive cycle, a child disclosed violence perpetration and child protection services had to be involved.

We also suggest enabling discussion of lived experiences without the need to disclose personal stories. In our work the trauma app it was stated, before the work commenced, that the young people would not have to share their own experiences of trauma as recounting their traumatic experiences may have led to re-traumatization or secondary traumatization of others in the group (Motta, 2008). In consultation with a child psychiatrist, the research team constructed case vignettes based on common traumatic events and post-traumatic stress symptoms. The purpose of this was to fulfill the trauma normalization aspect of the manualized intervention, by including ‘trauma stories’ in the app. With regard to the working process, the cases allowed the young people to discuss different traumatic experiences on a general level and we recommend integrating case vignettes into participatory research processes.

### Participation in Interviews

*Apply structure to interviews* Structured interviews have generally tended to enhance the quality of the interviewer’s performance. Our group utilizes the guidelines from the National Children’s Advocacy Center (2019). These include providing a safe interview setting, preparing the child in advance, and informing the child about the interview and the voluntary nature of it (e.g., “If you would like to stop, you can tell me or say *stop*, or you can show me with your hand like this”). In this phase, children are informed about the purpose of the interview, encouraged to talk only about things that have actually happened, to correct the interviewer when they are wrong, and to tell the interviewer if the child does not know or understand something. These conventions are also practiced together with the child in a non-intrusive manner. The guidelines include using a continuum of questions to ensure that the child is helped to give as complete and accurate a statement as possible, starting with open-ended questions and then focused narrative requested and detail-focused questions. When the interview draws to a close, the interviewer acknowledges the child’s participation and turns the conversation towards something neutral before ending the interview.

*Conduct interviews side-by-side* Interviewing in this way has been shown to be better than face-to-face, as sitting side-by-side is perceived as less didactic, less interrogative and less threatening. Side-by-side interviews also help to establish a working alliance. During the last 10 years our research group has been using and evaluating the computer-assisted interview In My Shoes for conducting interviews with children aged 3–5 years. In My Shoes is an interactive method, where the child and interviewer use the software together in a triadic interview. While not carrying out the actual interview, the computer program provides a visual aid, as well as structure, a shared external focus and tools for expressing emotional and relational experiences. This triadic setting has been particularly important for shy children, as it helped them increase their verbal communication during the rapport-phase of the interview (Fängstrom et al., 2017). In general, side-by-side interviews also improve the cognitive capacity of the youngest children compared to vis-à-vis, as eye contact has been shown to have a negative impact on elicitation and processing (Doherty-Sneddon & Phelps, 2005).

*Use pictures and symbols* Children are helped more than adults by picture- and symbol-supported conversations. One reason for this is that their memory retrieval strategies and capacities are still developing (Schwenck et al., 2009) and pictures can function as a memory aid or cue (Hamond & Fivush, 1991). In My Shoes includes stylized icons of emotions, people, speech, thoughts, sensations and places. Our experience is that these icons have served both as a facilitator for children to communicate their experiences and emotions and as a prompt for the interviewer to ask about various aspects of the children’s experiences (Fängstrom & Eriksson, 2020). Our research group has shown that interviews conducted through In My Shoes are both as accurate and complete as a standard forensic verbal interview, regarding children’s recall of factual information (Fängström et al., 2016).

The need for symbols and pictures is particularly important to consider in conversations with children who have language or communication difficulties as they can serve as an alternative way of communicating (Blackstone et al., 2007). Regrettably, when a child communicates with other means than spoken language they often are passive researched objects instead of active participators (Dindar et al., 2017; Tisdall & Kay, 2017). Even though they have vital experiences and insights, share the right to make their voices heard and, according to our experiences, *want* to make their voices heard (Fäldt et al., 2022), they are often excluded from being involved in the research process and even participating in studies (Tager-Flusberg & Kasari, 2013; Tisdall & Kay, 2012). This may be partly explained by the fact that including children with complex communication needs puts extra demands on the researcher. They must ensure that the child can give a valid informed consent (Stalker, 1998) and facilitate participation without influencing the child’s communication. There is also a tendency for communication partners to ‘interpret’ a child with complex communication needs. This creates the need for the child to challenge and correct these interpretations, which, in turn, is difficult and demanding for the child (Grove et al., 1999). An additional challenge is when the child shows signs of echolalia, i.e., repetitive vocalizing in response to a communication partner’s utterance, as echolalia can sometimes be a communicative signal that needs to be taken into account.

The most important preconditions to facilitate the participation of children with complex communication needs are: (i) responsive communication, including sensitivity to all the child’s communicative signals within the interview and the analysis; (ii) flexible use of different augmentative and alternative communication strategies, personalized to the specific needs of the participating child; and (iii) interviewers with experience communicating with individuals with complex communication needs.

### Participation in Co-produced Research Activities

*Do not underestimate the importance of the practical details* Existing knowledge on participatory research with children has highlighted the importance of the practical details, including neutral location, setting, day and time, breaks, and refreshments (Horgan & Martin, 2021; Preston et al., 2019). For the first workshop, a small conference room in a centrally located hotel was booked and the work was scheduled at the weekend. The young people expressed the wish that future workshops would also be held in the conference room rather than, for example, at the university. Regular breaks with refreshments and non-project conversations, the timing of which were decided by the youth, were highlighted as an important part of the whole experience.

*Build in enough time for the working process* Conducting research *with* children and young people differs from conducting research *on* or *about* them. Make sure there is enough time to actually do the work together. This means, for example, setting aside time for continuous joint reflections on the process—and enough time to take the reflections on board and adjust accordingly. Having enough time set aside also facilitates the higher level of flexibility that is often necessary on the part of the academic researchers.

*Facilitate communication* Not all children and young people prefer, or have access to, email. Opening up the possibility of communicating through other channels—such as text messaging, chat programs or phone calls—can help to enable each young person to be more involved between in-person contacts. A chat group allows for quick communication, but also requires everyone to be comfortable sharing their contact details with each other. Make sure to get approval for this in advance. In addition to this, be prepared that young people may not always have the time and willingness to communicate during daytime hours (i.e., during most academic researchers’ working hours). It may be necessary to talk through the appropriate times to have contact, so that it is clear to both young people and researchers.

*Co-create your working methods* Early in the trauma app project, a collaborative workshop method called Design Studio (Kaplan, 2017), was successfully tested for creating a logo for the app. As the project progressed, adaptations to the method were co-created to enable the participation and involvement of all seven young people to their own desired level. One such example, suggested by one of the young people, was for them to do parts of the first step (idea generation) on their own in-between workshops. This allowed more time with the tasks and, for those young people who preferred it, allowed them to express themselves in writing. However, consideration needed to be given to the nature of the work that could be performed outside the safe context of a group meeting with a researcher present. It is for this reason, the home assignments tended to focus on app esthetics rather than trauma-focused content.

*Training as a means for power-sharing* Now that the app is developed, usability testing with other young people as research participants is being carried out. Shifting the roles of young people involved in research, from conducting research *with* them to research being conducted *by* them, implies new considerations of issues related to power and decision-making. However, it also provides opportunities to take the necessary steps towards power-sharing. One such example is to provide them with the necessary training to carry out the tasks (Kellett, 2005). The training, which should provide theoretical and practical knowledge on research ethics and methodology, will enable the young people to actively lead and carry out research tasks without the direct presence of the academic researchers. As agreed with the young people, the academic researchers in our project are taking a passive role during data collection while, of course, ready to support if needed.

### Contribution

Children’s time, schedules and other commitments must be respected, their participation acknowledged, and their contribution recognized whether that is through reimbursement in vouchers or other means.

*Be clear on roles and responsibilities* Be clear on what can and should be the responsibility of the researchers and the youth, respectively. For example, youth might spread information about the app usability study and solicit potential study participants, yet the informed consent procedure needs to be conducted by a trained researcher. Likewise, usability testing can be performed by youth, but if an unexpected event occurs, e.g., a young person reacts strongly to the content of the app, a pre-determined safety protocol needs to be activated and the responsible researcher immediately notified. In practice, this means the data collection for our project is conducted with a responsible researcher in the immediate vicinity, but not in the same room, so as not to disturb data collection independently conducted by the youth. All this needs to be clearly and transparently presented to the ethical review board. We have been successful in gaining understanding for our co-production designs, although the role of youth as co-researchers (who do not need to consent to research) versus other youth as study participants (who do need to consent) had to be clarified after questions from the board.

## Conclusions

In this paper, we have made the case for the direct engagement of children in research on different levels of participation. Reflecting on examples from our own research, we highlight a number of considerations. When giving a voice to children in research, it is important to remember that talking to children is different from talking to adults. Applying structure to interviews, conducting them side-by-side, and making use of pictures and symbols can all facilitate the process. When working *with* children on research projects, attention should be given to the working methods; they should be co-created, with importance placed on practical details and timing. It is likely that communication will need to be facilitated, particularly between in-person contacts. When engaging children with a particular lived experience they should not feel pressure to disclose their personal stories. Child-led research requires researchers to share power, which can be facilitated through training, while clarity on roles and responsibilities is essential. While we have gained a lot of experience over the years, we are still learning. As we try to do, we encourage researchers to take a norm-critical perspective when approaching child and family research—to consciously and consistently ask questions about *how* children, particularly structurally vulnerable children, can be involved.

## Data Availability

The data that support the findings reported in this paper are available from the first author upon reasonable request. Restrictions apply to some data due to privacy or ethical reasons.

## References

[CR1] Åkerström J (2014). “Participation is everything”: Young people’s voices on participation in school life.

[CR2] Baumtrog MD, Peach H (2019). They can’t be believed: Children, intersectionality, and epistemic injustice. Journal of Global Ethics.

[CR3] Blackstone SW, Williams MB, Wilkins DP (2007). Key principles underlying research and practice in AAC. Augmentative Alternative Communication.

[CR4] Bokstrom P, Fangstrom K, Calam R, Lucas S, Sarkadi A (2016). ‘I felt a little bubbly in my tummy’: Eliciting pre-schoolers’ accounts of their health visit using a computer-assisted interview method. Child: Care, Health and Development.

[CR5] Burroughs MD, Tollefsen D (2016). Learning to listen: Epistemic injustice and the child. Episteme.

[CR6] Cahill H, Dadvand B (2018). Re-conceptualising youth participation: A framework to inform action. Children & Youth Services Review.

[CR7] Carel H, Györffy G (2014). Seen but not heard: Children and epistemic injustice. The Lancet.

[CR8] Cody C (2017). ‘We have personal experience to share, it makes it real’: Young people’s views on their role in sexual violence prevention efforts. Children and Youth Services Review.

[CR9] Dahlberg A, Salari R, Fängström K, Fabian H, Sarkadi A (2022). Successful implementation of parenting support at preschool: An evaluation of Triple P in Sweden. PLoS ONE.

[CR10] Dindar K, Lindblom A, Kärnä E (2017). The construction of communicative (in)competence in autism: A focus on methodological decisions. Disability Society.

[CR11] Doherty-Sneddon G, Phelps FG (2005). Gaze aversion: A response to cognitive or social difficulty?. Memory & Cognition.

[CR12] ERIC. (2016). *International charter for ethical research involving children*. ERIC Charter. https://childethics.com/charter/

[CR13] Eriksson M, Broberg AG, Hultmann O, Chawinga E, Axberg U (2022). Safeguarding children subjected to violence in the family: Child-centered risk assessments. International Journal of Environmental Research and Public Health.

[CR14] Eriksson M, Näsman E (2008). Participation in family law proceedings for children whose father is violent to their mother. Childhood.

[CR15] Eriksson M, Näsman E (2012). Interviews with children exposed to violence. Children & Society.

[CR17] Facca D, Gladstone B, Teachman G (2020). Working the limits of “giving voice” to children: A critical conceptual review. International Journal of Qualitative Methods.

[CR18] Fäldt A, Klint F, Warner G, Sarkadi A (2022). Experiences of children with disabilities during the COVID-19 pandemic in Sweden: A qualitative interview study. BMJ Paediatrics Open.

[CR19] Fängström K, Bokstrom P, Dahlberg A, Calam R, Lucas S, Sarkadi A (2016). In My Shoes—Validation of a computer assisted approach for interviewing children. Child Abuse and Neglect.

[CR20] Fängstrom K, Eriksson M (2020). The feasibility of the In My Shoes computer assisted interview for eliciting evaluative content in interviews with young children. Children and Youth Services Review.

[CR21] Fängstrom K, Salari R, Eriksson M, Sarkadi A (2017). The computer-assisted interview In My Shoes can benefit shy preschool children’s communication. PLoS ONE.

[CR22] Fängström K, Sarkadi A, Lucas S, Calam R, Eriksson M (2017). “And they gave me a shot, it really hurt”—Evaluative content in investigative interviews with young children. Children and Youth Services Review.

[CR23] Fricker M (2007). Epistemic injustice: Power and the ethics of knowing.

[CR24] Gräfe AW, Englander M (2022). Listening to the social world of the child: A phenomenological proposal. Barn–forskning Om Barn Og Barndom i Norden.

[CR25] Grove N, Bunning K, Porter J, Olsson C (1999). See what I mean: Interpreting the meaning of communication by people with severe and profound intellectual disabilities. Journal of Applied Research in Intellectual Disabilities.

[CR26] Hamond NR, Fivush R (1991). Memories of mickey mouse—Young-children recount their trip to Disneyworld. Cognitive Development.

[CR27] Hart, R. A. (1992). *Children’s participation: From tokenism to citizenship*. https://www.unicef-irc.org/publications/pdf/childrens_participation.pdf

[CR28] Hart RA, Reid BJA, Nikel J, Simovska V (2008). Stepping back from “the ladder”: Reflections on a model of participatory work with children. Participation and learning.

[CR29] Heimer M, Näsman E, Palme J (2018). Vulnerable children’s rights to participation, protection, and provision: The process of defining the problem in Swedish child and family welfare. Child & Family Social Work.

[CR30] Horgan D, Martin S (2021). Children’s research advisory groups: Moving from adult-research agendas to co-creation with children. Child and youth participation in policy, practice and research.

[CR31] Houghton C (2015). Young people’s perspectives on participatory ethics: Agency, power and impact in domestic abuse research and policy-making. Child Abuse Review.

[CR32] Houghton C, Holt S, Øverlien C, Devaney J (2018). Voice, agency, power: A framework for young survivors’ participation in national domestic abuse policy-making. Responding to domestic violence: Emerging challenges for policy, practice and research in Europe.

[CR33] Iversen C (2012). Recordability: Resistance and collusion in psychometric interviews with children. Discourse Studies.

[CR34] Iversen C (2019). Beyond accessing information: Claiming to understand in child social welfare interviews. British Journal of Social Psychology.

[CR35] James A, Jenks C, Prout A, Jenks C (1998). Theorizing childhood. Childhood: Critical concepts in sociology.

[CR36] Kaminski JW, Valle LA, Filene JH, Boyle CL (2008). A meta-analytic review of components associated with parent training program effectiveness. Journal of Abnormal Child Psychology.

[CR37] Kaplan K (2017). Facilitating an effective design studio workshop.

[CR38] Kay E, Tisdall M (2012). The challenge and challenging of childhood studies? Learning from disability studies and research with disabled children. Children & Society.

[CR39] Kellett M (2005). How to develop children as researchers.

[CR40] Kellett M (2010). Rethinking children and research: Attitudes in contemporary society.

[CR41] Kellett M, Forrest R, Dent N, Ward S (2004). ‘Just teach us the skills please, we’ll do the rest’: Empowering ten-year-olds as active researchers. Children & Society.

[CR42] Kennan D, Brady B, Forkan C (2019). Space, voice, audience and influence: The Lundy model of participation (2007) in child welfare practice. Practice.

[CR43] Larsson I, Staland-Nyman C, Svedberg P, Nygren JM, Carlsson I-M (2018). Children and young people’s participation in developing interventions in health and well-being: A scoping review. BMC Health Services Research.

[CR44] Liabo K, Ingold A, Roberts H (2018). Co-production with “vulnerable” groups: Balancing protection and participation. Health Science Reports.

[CR45] Lundy L (2007). ‘Voice’is not enough: Conceptualising article 12 of the United Nations convention on the rights of the child. British Educational Research Journal.

[CR46] Lundy L, McEvoy L, Kilkelly U, Lundy L (2017). Children’s rights and research processes: Assisting children to (in)formed view. Children’s rights.

[CR47] Lygnegård F, Thell M, Sarkadi A (2023). Adolescent co-researchers identified the central role of social media for young people during the pandemic. Acta Paediatrica.

[CR48] McMellon C, Tisdall EKM (2020). Children and young people’s participation rights: Looking backwards and moving forwards. The International Journal of Children’s Rights.

[CR49] Motta RW (2008). Secondary trauma. International Journal of Emergency Mental Health.

[CR50] Mullender A, Hague G, Imam U, Kelly L, Malos E, Regan L (2002). Children’s perspectives on domestic violence.

[CR51] National Children’s Advocacy Center (2019). National Children’s Advocacy Center’s child forensic interview structure.

[CR52] Pérez-Aronsson A, Thell M, Lampa E, Löfving SG, Tökés A, Torakai N, Ibrahim K, Aljeshy R, Warner G (2022). Adaptation of the trauma group intervention ‘Teaching Recovery Techniques’ for online delivery: A participatory design and usability study. Internet Interventions.

[CR53] Preston J, Stones SR, Davies H, Preston J, Phillips B (2019). How to involve children and young people in what is, after all, their research. Archive of Disease in Childhood.

[CR54] Prout A, James A, James A, Prout A (2015). A new paradigm for the sociology of childhood? Provenance, promise and problems. Constructing and reconstructing childhood.

[CR55] Rahmqvist J, Wells M, Sarkadi A (2013). Conscious parenting: A qualitative study on Swedish parents’ motives to participate in Triple P, the Positive Parenting Program. Journal of Child and Family Studies.

[CR56] Robinson L, Adair P, Coffey M, Harris R, Burnside G (2016). Identifying the participant characteristics that predict recruitment and retention of participants to randomised controlled trials involving children: A systematic review. Trials.

[CR57] Salari R, Wells MB, Sarkadi A (2014). Child behaviour problems, parenting behaviours and parental adjustment in mothers and fathers in Sweden. Scandinavian Journal of Public Health.

[CR58] Sammons HM, Wright K, Young B, Farsides B (2016). Research with children and young people: Not on them. Archives of Disease in Childhood.

[CR59] Sampaio F, Sarkadi A, Salari R, Zethraeus N, Feldman I (2015). Cost and effects of a universal parenting programme delivered to parents of preschoolers. European Journal of Public Health.

[CR60] Sandland R (2017). A clash of conventions?: Participation, power and the rights of disabled children. Social Inclusion.

[CR61] Sarkadi A, Dahlberg A, Fängstrom K, Warner G (2020). Children want parents to ask for permission before ‘sharenting’. Journal of Paediatrics and Child Health.

[CR62] Sarkadi A, Lampa E, Warner G (2022). Promoting an understanding of forced migration among host country children and exploring their views on refugee children’s needs. Journal of Immigrant and Minority Health.

[CR63] Sarkadi A, Sahlin Torp L, Perez-Aronsson A, Warner G (2021). Children’s expressions of worry during the COVID-19 pandemic in Sweden. Journal of Pediatric Psychology.

[CR64] Sarkadi A, Thell M, Jirblom K (2023). Perceptions of the COVID-19 pandemic as demonstrated in drawings of Swedish children aged 4–6 years. Acta Paediatrica.

[CR65] Sarkadi A, Warner G, Salari R, Fängström K, Durbeej N, Lampa E, Baghdasaryan Z, Osman F, Gupta Löfving S, Perez Aronsson A, Feldman I, Ssegonja R, Calam R, Bjärtå A, Leiler A, Rondung E, Wasteson E, Oppedal B, Keeshin B (2020). Evaluation of the Teaching Recovery Techniques community-based intervention for unaccompanied refugee youth experiencing post-traumatic stress symptoms (Swedish UnaccomPanied yOuth Refugee Trial; SUPpORT): Study protocol for a randomised controlled trial. Trials.

[CR66] Schwenck C, Bjorklund DF, Schneider W (2009). Developmental and individual differences in young children’s use and maintenance of a selective memory strategy. Developmental Psychology.

[CR67] Shier H (2001). Pathways to participation: Openings, opportunities and obligations. Children & Society.

[CR68] Shier H, Berson IR, Berson MJ, Gray C (2019). An analytical tool to help researchers develop partnerships with children and adolescents. Participatory methodologies to elevate children’s voice and agency.

[CR69] Skyrme SL, Woods S (2018). Researching disabled children and young people’s views on decision-making: Working reflexively to rethink vulnerability. Childhood.

[CR70] Stalker K (1998). Some ethical and methodological issues in research with people with learning difficulties. Disability & Society.

[CR71] Tager-Flusberg H, Kasari C (2013). Minimally verbal school-aged children with autism spectrum disorder: The neglected end of the spectrum. Autism Research.

[CR72] Tisdall E, Kay M (2012). The challenge and challenging of childhood studies? Learning from disability studies and research with disabled children. Children & Society.

[CR73] Tisdall E, Kay M (2017). Conceptualising children and young people’s participation: Examining vulnerability, social accountability and co-production. The International Journal of Human Rights.

[CR74] UN. (1989). *Convention on the rights of the child*. UN Doc. A/RES/44/25. http://www2.ohchr.org/english/law/pdf/crc.pdf

[CR75] Warner G, Durbeej N, Salari R, Fängström K, Lampa E, Baghdasaryan Z, Osman F, Löfving SG, Aronsson AP, Feldman I, Sampaio F, Ssegonja R, Bjärtå A, Rondung E, Leiler A, Wasteson E, Calam R, Oppedal B, Keeshin B, Sarkadi A (2020). Evaluation of the teaching recovery techniques community-based intervention for accompanied refugee children experiencing post-traumatic stress symptoms (Accompanied refugeeS In Sweden Trial; ASsIST): Study protocol for a cluster randomised controlled trial. British Medical Journal Open.

[CR76] Wells MB, Sarkadi A, Salari R (2016). Mothers’ and fathers’ attendance in a community-based universally offered parenting program in Sweden. Scandinavian Journal of Public Health.

